# Effects of ibrutinib and venetoclax on the expression of immune checkpoint molecules in leukemic blasts of patients with acute lymphoblastic leukemia

**DOI:** 10.1016/j.bbrep.2025.102045

**Published:** 2025-05-12

**Authors:** Armin Dozandeh-Jouybari, Fatemeh Mousavi-Mirkalaei, Saeid Taghiloo, Hossein Karami, Mohammad Naderisorki, Ehsan Zaboli, Mohammad Eslami-Jouybari, Tohid Kazemi, Hossein Asgarian-Omran

**Affiliations:** a Department of Immunology, School of Medicine, Mazandaran University of Medical Sciences, Sari, Iran; b Immunology Research Center, Tabriz University of Medical Sciences, Tabriz, Iran; c Thalassemia Research Center (TRC), Hemoglobinopathy Institute, Mazandaran University of Medical Sciences, Sari, Iran; d Gastrointestinal Cancer Research Center, Non-Communicable Diseases Institute, Mazandaran University of Medical Sciences, Sari, Iran; e Department of Hematology and Oncology, Imam Khomeini Hospital, Mazandaran University of Medical Science, Sari, Iran; f Department of Immunology, Faculty of Medicine, Tabriz University of Medical Sciences, Tabriz, Iran

**Keywords:** ALL, Ibrutinib, Venetoclax, Immune checkpoint molecules, TGF-β

## Abstract

**Background:**

In recent decades, targeted therapy using small molecule inhibitors (SMI) have been shown very promising results in the treatment of a variety of solid and hematopoietic malignancies. However, their exact mechanisms, especiallay on the evasion strategies of tumor cells from the host immune system are not fully understood. The current study investigates the effects of two SMIs, ibrutinib and venetoclax, on the expression of inhibitory immune checkpoint molecules in patients with acute lymphoblastic leukemia (ALL).

**Methods:**

Leukemic cells were isolated from 20 patients with ALL by magnetic activated cell sorting (MACS) technique. Isolated leukemic cells were cultured and treated by ibrutinib and venetoclax for 48 h. Cell viability and apoptosis were monitored through MTT and flow cytometry assays, respectively. The mRNA expression levels of checkpoint molecules PD-L1, galectin-9, CD200, CD155, CD47, and anti-inflammatory cytokine TGF-β were determined by Real-Time PCR method.

**Results:**

The purity of MACS-isolated ALL leukemic cells was >98% as determined by flow cytometry. Following treatment, the proliferation of leukemic cells was significantly decreased and the apoptosis rate was significantly increased, which was more remarkable for venetoclax. Moreover, treatment of leukemic cells with ibrutinib and venetoclax showed alterations in the mRNA expression of immune checkpoint inhibitory ligands and TGF-β.

**Conclusion:**

Our results indicated that small molecule inhibitors not only hinder proliferation and enhance apoptosis, but also affect the expression of inhibitory immune checkpoint ligands. By elucidating the precise underlying mechanisms, these drugs could emerge as promising therapeutic options, particularly in the context of combination therapy for ALL.

## Highlights

• Higher cytotoxicity of ibrutinib and venetoclax in ALL compared to vincristine.
• More apoptosis rate for venetoclax in ALL compared to ibrutinib and vincristine.
• Alterations in the mRNA expression of PD-L1, Gal-9, CD47, CD200, CD155 and TGF-β.
• Small molecule inhibitors affect the expression of immune inhibitory molecules.
• SMIs could emerge as promising therapeutic options for combination therapy of ALL.

## 1 Introduction

Acute lymphoblastic leukemia (ALL) is the most prevalent malignancy among children which is characterized by maturation arrest and unregulated growth of immature B and T lineages, and their accumulation in the bone marrow and peripheral blood [1,2]. Its prevalence is different based on age, sex, and race [3] with peak incidences in children between the ages of 1–4 years old, and in adults 55 years of age or beyond [4]. The estimated yearly incidence of ALL worldwide is 1–5 cases/100,000 people and males are more likely than females to develop ALL, with a ratio of 1.2:1 [5]. Despite advances in ALL treatment, significant challenges remain, particularly in low- and middle-income countries. Chemotherapy, which is still the mainstay of treatment, has many limitations [6]. It typically involves four stages: induction, consolidation, intensification, and maintenance. Non-specific and unfavorable side effects of chemotherapeutic drugs cause treatment resistance and disease relapse in ALL patients, which s researchers to discover new targeted therapies with reduced toxicity and better cure rates [7,8]. In this regard, targeted therapies including blinatumomab as a bispecific T cell engager, inotuzumab ozogamicin as an antibody-drug conjugate, and tisagenlecleucel as a CAR-T cell were approved for the treatment of ALL patients [9]. Small molecule inhibitors (SMI) are another category of targeted drugs, which is a new generation of chemotherapy drugs. Some of their advantages are small size (≤500Da) lead to target intracellular and cell surface molecules, pharmacokinetic properties, cost-effectiveness, and amenability to oral administration, patient compliance, drug storage, transportation, and the possibility of combination with other therapeutic strategies [10,11]. Bruton tyrosine kinase (BTK) and B-cell lymphoma 2 (Bcl-2) are two key molecules involved in B-ALL cell survival and immune evasion mechanisms [12,13]. Venetoclax and ibrutinib are novel SMIs that target Bcl-2 and BTK, respectively [13,14]. Using the mentioned treatment strategies for ALL patients resulted in increasing 5-years overall survival (OS) by up to 90% in children, but OS is still less than 45% in adults [7]. Despite the progresses in ALL treatment by targeted therapy, advanced treatment options are not available worldwide and as a result, a sizeable portion of patients, especially adults, still do not benefit from it [6].

On the other hand, ALL blast cells apply various mechanisms to evade from the host immune system. These mechanisms include reducing immunogenic antigens, disrupting T cell activation due to the absence of co-stimulatory molecules, decrease in neoantigens construction resulting from the minimal burden of mutations, increasing anti-apoptotic proteins such as Bcl-2, overproduction of TGF-β and IL-10, and also expression of immunosuppressive ligands such as programmed death-ligand 1 (PD-L1) [15], galectin-9 (Gal-9) [16], CD155 [17], CD200 [18] and CD47 [19]. However, the exact signaling pathways by which Bcl-2 and BTK inhibition influence immune escape mechanisms in ALL remain unclear. This study aims to investigate the impacts of Bcl-2 and BTK inhibition on the expression of inhibitory checkpoint ligands and cytokines in ALL leukemic blasts.

## 2 Methods and materials

### 2.1 Study population and bone marrow samples

In this study, 20 patients were incorporated after definitive diagnosis of ALL. Diagnosis was done by hematology-oncologist clinicians based on the WHO criteria including examination of complete blood count, bone marrow smear, and immunophenotyping analysis by flow cytometry. None of the participants were undergone treatment before sampling. Bone marrow samples were taken after obtaining written informed consent from all participants. The hematological information of patients are represented in Table 1. This study was approved to be ethically admissible by the Ethical Committee of Mazandaran University of Medical Sciences (IR.MAZUMS.IMAMHOSPITAL.REC.1401.15064).

**Table 1:** Demographic & hematologic information of ALL patients.

Code	Sex	Age	WBC (∗10^3^/μL)	Hb (g/dL)	PLT/(∗10^3^/μL)	CD10 (%)	CD19 (%)	CD20 (%)	CD34 (%)	HLA-DR (%)	Subtype
**ALL-1**	M	31	58.7	7.6	21	93	92	1.3	90	91	Early Pre-B
**ALL-2**	F	2	66.8	7.1	14	95	95	23	73	89	Pre-B
**ALL-3**	M	33	49.8	12	33	97	97	79	95	95	Pre-B
**ALL-4**	M	12	52.7	4	83	0.2	92.1	0.1	91.7	91.8	Pro-B
**ALL-5**	F	2	25.3	9.8	89	84.4	86.2	3.6	0.4	88.4	Early Pre-B
**ALL-6**	M	NA	NA	NA	NA	88.9	94.3	23.8	53.7	92.5	Early Pre-B
**ALL-7**	M	31	18.3	8.8	18	80	7.7	0.1	85	95	Pre-B
**ALL-8**	F	1	76	9.7	144	0.1	86.1	0.1	88.9	88.1	Pro-B-
**ALL-9**	F	30	256	9.5	127	92.3	95.9	14.2	93.1	96.5	Early Pre-B
**ALL 10**	F	9	4.7	7.5	74	83.7	90.9	4.1	3.9	91.1	Early Pre-B
**ALL-11**	F	14	245	6.5	57	95	95	23	65	80	Pre-B
**ALL-12**	F	3	5	3.8	38	91	91	1.2	74	88	Early Pre-B
**ALL-13**	M	4	9.9	8.4	7	95	94	2.1	16	34	Early Pre-B
**ALL-14**	M	14	147	5.7	8	98	98	39	74	97	Pre-B
**ALL-15**	F	3	24.5	10.2	22	0.6	93	1.5	43	94	Early Pro-B
**ALL-16**	F	40	91	6.6	167	0.1	72	1.1	44	84	Pro-B
**ALL-17**	M	19	67	6.9	57	90	84	0.2	88	98	Early Pre-B
**ALL-18**	M	18	19.5	4.2	100	40	97	0.5	98	99	Early Pre-B
**ALL-19**	M	70	1.7	9.4	163	44	46	4.1	44	57	Early Pre-B
**ALL-20**	M	55	12.5	8.9	27	87	92	0.8	85	97	Early Pre-B

ALL: acute lymphoblastic leukemia, M: male, F: female, WBC: white blood cell, Hb: hemoglobin, PLT: platelet, CD: cluster of differentiation, NA: not available.

### 2.2 Chemical compounds and reagents

Venetoclax and ibrutinib were bought from Cayman Chemical Company (Michigan, USA). Both SMIs were dissolved in cell culture-grade dimethyl sulfoxide and then aliqouted. Vincristine was also obtained from KOCAK company (Istanbul, Turkey).

### 2.3 Isolation of bone marrow CD19^+^ leukemic B-cells

Bone marrow mononuclear cells were isolated from bone marrow aspiration samples by density gradient centrifugation on Ficoll-Histopaque (Cytiva, Uppsala, Sweden). Isolated cells were washed twice with RPMI-1640 culture medium (Biosera, Nuaille, France) supplemented with penicillin (100 IU/mL) and streptomycin (100 μg/mL) (Biosera, Nuaille, France). The viability of isolated cellsm was >98 % as determined by trypan blue staining. To isolate CD19^+^ leukemic B-cells by magnetic-activated cell sorting (MACS) method, the cell pellet was resuspended in MACS buffer (PBS 0.15 M containing 0.5 % BSA, and 2 mM EDTA), and then passed through the 70 μm pre-separation filter (Miltenyi Biotec, Bergisch Gladbach, Germany) to remove clamp and debris. CD19^+^ B-cells were positively isolated by a magnetic beads-conjugated anti-CD19 mAbs kit (Miltenyi Biotec, Bergisch Gladbach, Germany) according to the manufacturer's instructions. The purity of isolated CD19^+^ leukemic cells was checked by two-color flow cytometry analysis using CD10-APC and CD34-PE antibodies (Becton Dickinson, California, USA) by a Beckman-Coulter flow cytometry istrument.

### 2.4 Determination of optimal concentration of drugs

To obtain half maximal inhibitory concentration (IC50) of applied drugs, leukemic cells were seeded in a flat-bottom 96 culture plate (2 × 10^5^/well) in 200 μL RPMI-1640 medium, supplemented with penicillin (100 IU/mL), streptomycin (100 μg/mL) and 10% heat-inactivated fetal bovine serum. The cells were treated with various concentrations of ibrutinib (0.3–20 μM), venetoclax (0.1–12.5 nM), and vincristine (0.4–100 nM), and then incubated at 37 °C in a humidified incubator containing 5% CO2 (Tuttlingen, Germany) for 24 h, 48 h and 72 h. MTT assay was done. After incubation, 20 μL of 5 mg/ml MTT solution was added to each well and incubated for 4 h at 37 °C. After that, the microplates were centrifuged for 10 min at 300g, the supernatants were disposed and 150 μL of DMSO was added to each well. Microplates were shaken for 40 min to dissolve the formazan crystals, and then the absorbance was measured at 570 nm in a 720 nm reference wavelength on a plate reader (Synergy H1 BioTek, Winooski, USA). Cell-free culture medium was considered as blank, and untreated cells were designated as control. To obtain the final optical density, the optical density difference between wavelengths 570 and 720 nm was computed and subtracted from the optical density of the growth media without cells. Optimal concentration was calculated for all drugs using GraphPad Prism version 10 (San Diego, USA) curve fitting software.

### 2.5 Cell culture and treatment

Isolated ALL leukemic cells were treated with ibrutinib, venetoclax, and vincristine as a FDA-approded chemotherapeutic drug. Untreated cells were considered as control group. Leukemic cells were seeded in a flat-bottom 96-well microplate (2 × 10^5^/well) in 200 μL complete RPMI-1640 medium containing penicillin, streptomycin, and 10% FBS for 48h to measure cell viability. In addition, for cell apoptosis assay and RNA extraction, isolated leukemic cells were cultured in a flat-bottom 6-well culture plate (3 × 10^6^/well) in 3 ml complete medium.

### 2.6 Cell apoptosis assay

After seeding the isolated leukemic cells in 6-well plates, the cells were treated for 48h with optimal concentration of ibrutinib, venetoclax, and vincristine, and then apoptosis assay was performed using Annexin V/Propidium iodide (PI) apoptosis kit (Immunostep, Salamanca, Spain) according to the manufacturer's instruction. Briefly, a number of 1 × 10^6^ leukemic cells were washed twice with PBS and resuspended in 100 μL binding buffer, and incubated with 1 μL Annexin V-FITC and 1 μL PI for 15 min at room temperature in the dark. After incubation, 900 μL binding buffer was added to each sample and were scanned by a Partec PAS flow cytometer system (Partec GmBH, Munster, Germany). Unstained cells were also applied for optimal gating and to subtract the background staining.

### 2.7 RNA extraction and cDNA synthesis

Following treatment of leukemic cells with drugs in 6-well plates at 3 × 10^6^ cells/well for 48 h, total RNA was extracted using the Denazist Asia kit (Mashhad, Iran) according to the manufacturer's instructions. Extracted RNA was quantitatively and qualitatively confirmed using a nano-spectrophotometer (WPA, Cambridge, England) and electrophoresis (Bio-Rad, UK), respectively. Complementary DNA (cDNA) was reverse-transcribed from 1 μg total RNA in a 20 μl reaction mixture using the Yekta-Tajhiz cDNA synthesis kit (Tehran, Iran) in two steps. First, 1 μg of total RNA, 1 μl random hexamer primer, and DEPC-treated water were added to the microtube with 13.5 μl final volume and incubated at 70 °C for 5 min. After that, 4 μl of 5X first-strand buffer, 1 μl dNTP 10 mM, 0.5 μl RNasein 40 u/μl, and 1 μl M-MLV RT enzyme were added and incubated at 37 °C for 1 h, and finally at 70 °C for 5 min.

### 2.8 Semi-quantitative Real-Time polymerase chain reaction

Semi-quantitative Real-Time PCR (qRT-PCR) was done in a StepOne Real-Time PCR System (Applied Biosystems, Foster City, CA, USA) using SYBR green as the detection dye (Ampliqon, Copenhagen, Denmark). Primers were obtained from Metabion International AG (Planegg, Germany) and their efficiencies were confirmed by serial dilution standard curves. Primer pairs sequences were shown in Table 2. The PCR reactions were set as following steps: initial denaturation at 95 °C for 15 min followed by 45 cycles of denaturation at 94 °C for 30 s, annealing at 60 °C (for Gal-9, PD-L1, CD200, CD47, and β-actin) 60.5 °C (for CD155 and TGF-β genes) for 30 s and extension at 72 °C for 30 s. A melting curve analysis by Linreg software was performed for each run to verify that there were no primer dimers and that the amplification curves were specific. Finally, the relative mRNA levels of Gal-9, PD-L1, CD200, CD47, CD155, and TGF-β were normalized to β-actin and the relative expression of each molecule was calculated using the 2^−ΔΔCt^ method.

**Table 2:** Primer sequences used for Real-Time PCR.

Gene	Primers (5′-3′)	Product Size (bp)
**Galectin-9**	F: CAGTGCTCAGAGTTCCACA R: TGAGGCAGTGAGCTTCACAC	118
**PD-L1**	F: CTATGGTGGTGCCGACTACAA R: CTGCTTGTCCAGATGACTTCG	159
**CD200**	F: GTCTGTTACCAGCATCCT R: CTTAGCAATAGCGGAACTG	147
**CD155**	F: GGACGGCAAGAATGTGAC R: CCAGTTGTTATCATAGCCAGAG	124
**CD47**	F: TTTCGTCCCAGGTGAATA R: CCACAGCGAGGATATAGG	173
**TGF-β**	F: CGCCAGAGTGGTTATCTT R: TAGTGAACCCGTTGATGTC	147
**β -actin**	F: CCTTCCTGGGCATGGAGTCCT R: TGGGTGCCAGGGCAGTGAT	174

F: forward primer, R: reverse primer, bp: base pair.

### 2.9 Statistical analysis

Statistical analysis were performed with GraphPad Prism 10 (San Diego, CA, USA). Quantitative data are expressed as mean ± SEM. The Kolmogorov-Smirnov test and Shapiro-Wilk tests were employed to determine the normality of the distribution of the obtained data, followed by one-way analysis of variance and a *t*-test. P-values less than 0.05 were considered statistically significant.

## 3 Results

### 3.1 Isolation of CD19^+^ leukemic cells and evaluating their cellular viability following treatment with ibrutinib, venetoclax, and vincristine

**Fig. 1:**
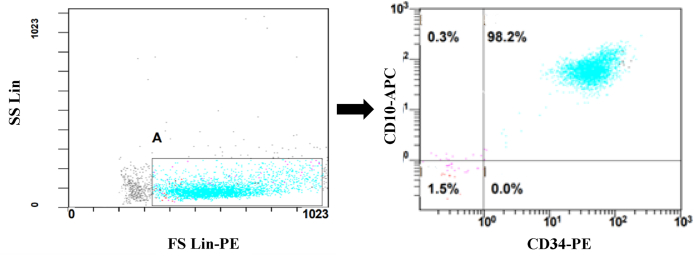
Purity determination of isolated ALL leukemic cells by flow cytometry. Magnetic cell activated sorted ALL leukemic CD19^+^ cells were staind with anti-CD10-APC and anti-CD34-PE and scanned by flow cytometry. The purity of isolated cells was determined to be >98%.

**Fig. 2:**
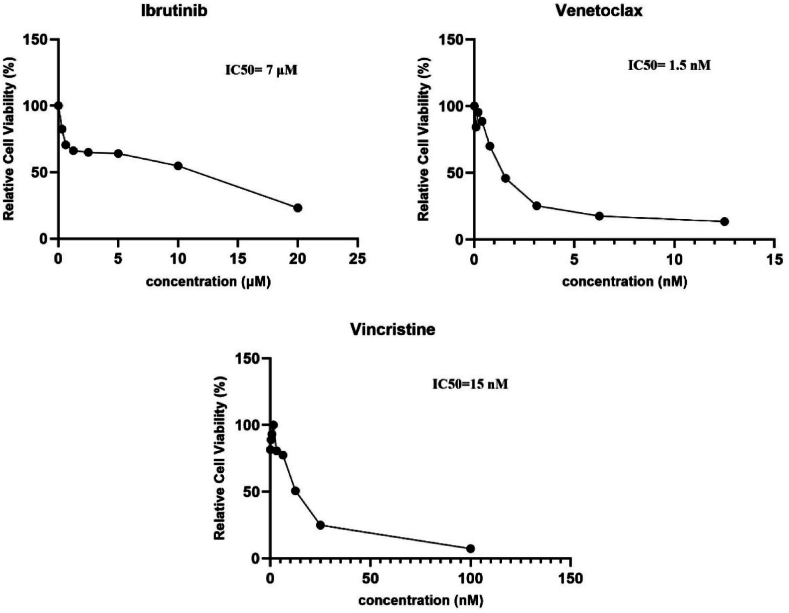
Determination of the half maximal inhibitory concentration (IC50) values of ibrutinib, venetoclax, and vincristine on ALL leukemic cells. Isolated leukemic cells were treated with increasing concentrations of ibrutinib, venetoclax, and vincristine for 48 h. Cell viability was measured by MTT assay. The IC50 values of ibrutinib, venetoclax, and vincristine were calculated to be 7 μM, 1.5 nM, and 15 nM, respectively.

Following the separation of CD19^+^ leukemic cells, two-color flow cytometry analysis revealed a purity percentage of >98% for leukemic cells (Fig. 1). To determine the IC50 concentration, different concentrations of ibrutinib, venetoclax, and vincristine were applied to leukemic cells during 24, 48, and 72 h of treatment and then cell viability was monitored by MTT assay. The 48-h treatment was shown to be more optimum than the 24 and 72 h treatments, which was chosen for subsequent experiments. Leukemic cells showed a dose-dependent decrease in viability after treatment with escalating doses of all drugs and the IC50 values were determined 7 μM, 1.5 nM, and 15 μM for ibrutinib, venetoclax, and vincristine, respectively (Fig. 2).

### 3.2 BTK and Bcl-2 inhibitors can cause proliferation inhibition in leukemic cells

**Fig. 3:**
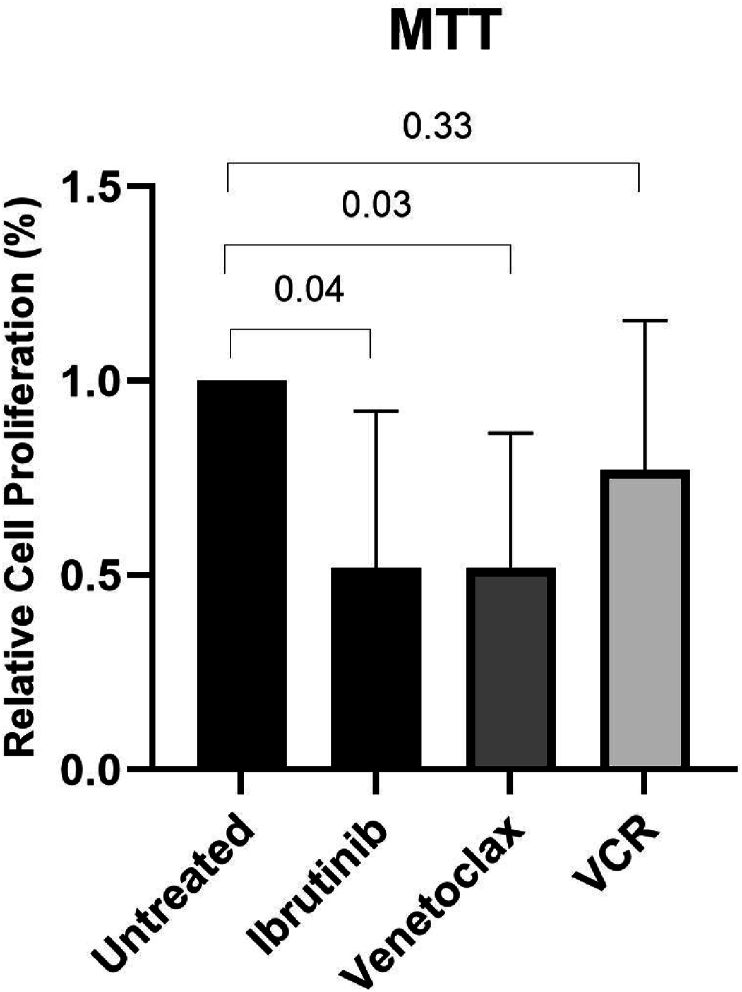
Inhibitory effects of small molecule inhibitors on the relative cell proliferation of ALL leukemic cells. Isolated leukemic cells were treated with ibrutinib, venetoclax, or vincristine for 48h. After that, the cell proliferation capacity was determined by MTT assay. After treatment with all drugs, a reduction in proliferation was seen in all groups, which was statistically significant for ibrutinib and venetoclax. Data are presented as mean ± SEM.

Following the determination of IC50 concentration, the anti-proliferative effect of ibrutinib, venetoclax, and vincristine were tested for each ALL sample after 48h incubation by MTT assay. Following treatment with all drugs, the proliferation of leukemic cells were significantly decreased, but the results were statistically significant for ibrutinib and venetoclax (p = 0.04 and p = 0.03, respectively). As represented in Fig. 3, despite the reduced proliferation, the results were not significant for vincristine group.

### 3.3 BTK and Bcl-2 inhibitors increased apoptosis in ALL leukemic cells

**Fig. 4:**
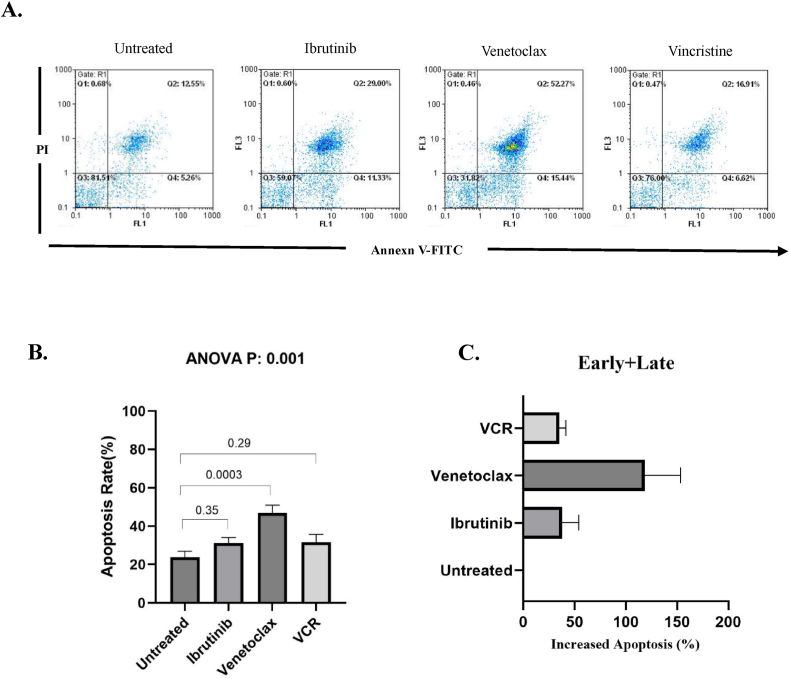
Effects of small molecule inhibitors on the apoptosis of isolated ALL leukemic cells. Isolated leukemic cells were cultured and treated with ibrutinib, venetoclax, and vincristine. After that, the apoptotic cells were detected by Annexin-V/PI staining assay via flow cytometry. A. A representative flow cytometric dot plot is shown. B and C. The percentage of apoptotic leukemic cells and the percentage of increased apoptosis rate in leukemic cells are represented. represented. One-way ANOVA with the Dunnett post hoc test were used for analyses.

To investigate the apoptosis rate, isolated ALL leukemic cells were treated with ibrutinib, venetoclax, and vincristine for 48 h. After that, the cells were collected and the level of apoptosis was measured using Annexin-V/PI staining by flow cytometry. Obtained results demonstrated increasing in the level of apoptosis after treatment with all drugs which showed strongly significant for venetoclax (p < 0.001) (Fig. 4a and b).

### 3.4 The effects of ibrutinib, venetoclax, and vincristine on the mRNA expression of immune checkpoint molecules in ALL leukemic cells

**Fig. 5:**
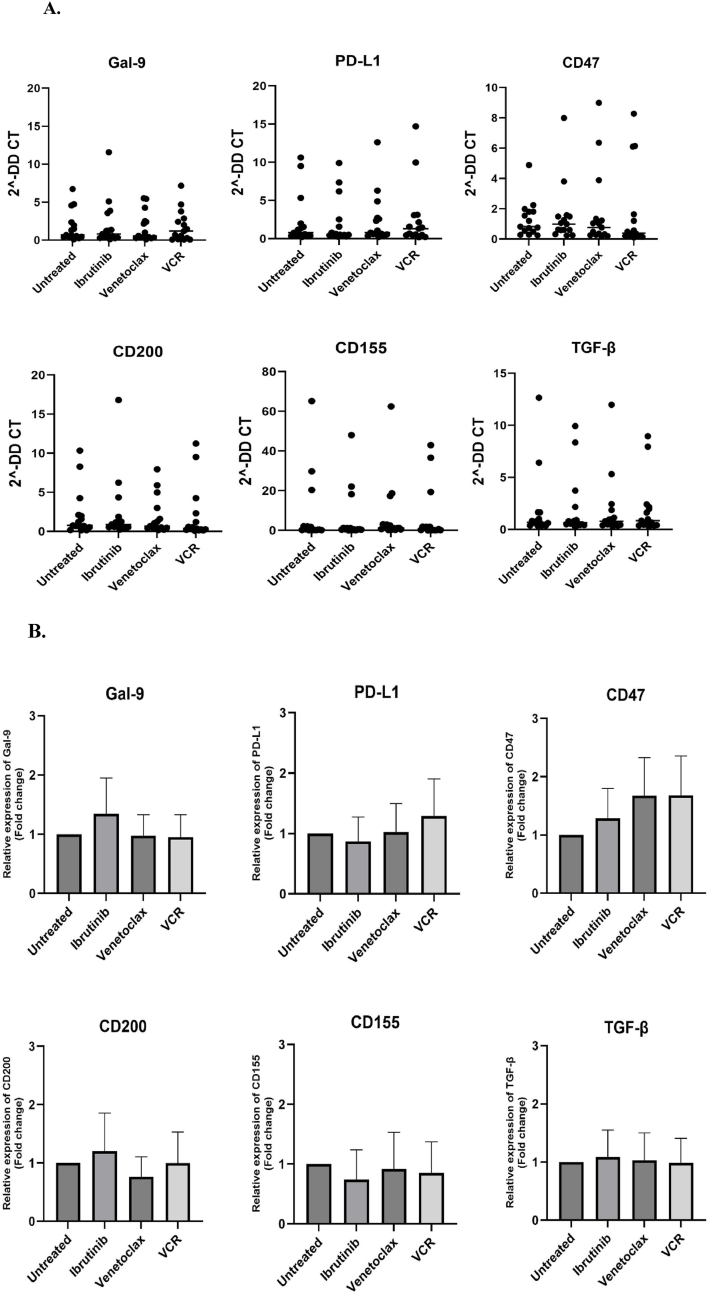
Effects of ibrutinib, venetoclax and vincristine on the mRNA expression of PD-L1, Gal-9, CD155, CD200, CD47, and TGF-β. Isolated leukemic cells were treated with ibrutinib, venetoclax, and vincristine. Gene expression results are represented as mean ± SEM (p < 0.05) of 2^−ΔΔCt^ after normalization with β-actin as an internal control. Both relative expression (A) and fold cange (B) are presented.

To elucidate the underlying immune evasion mechanisms in ALL leukemic cells, the effects of Bcl-2 and BTK inhibitors were assessed on the mRNA expression profile of immune checkpoint ligands PD-L1, CD200, CD155, Gal-9, and CD47, as well as the anti-inflammatory cytokine TGF-β, in leukemic cells. As shown in Fig. 5, the obtained results indicated that ibrutinib could reduce the expression of CD155 while increasing the mRNA expression level of Gal-9 and CD200. Moreover, venetoclax decreased CD200 expression, and the expression of PD-L1 was increased after treatment with vincristine. In addition, all tested drugs showed an increasing trend in CD47 mRNA expression. However, as indicated in Fig. 5, these differences were not statistically significant.

## 4 Discussion

Despite the recent advances in the strategies for treatment of ALL patients, there are still challenges that decrease the therapeutic efficacy and overall survival. One of the most significant of these challenges is the abilities of tumor cells to inhibit and divert the host anti-tumor immune responses. It has been well documented in previous reports that ALL leukemic cells employ various immune evasion mechanisms and signaling pathways which can lead to the immune system inhibition. These pathways showed crucial roles in the oncogenesis processes of ALL leukemic cells by increasing the survival and proliferation rate of leukemic [20]. In recent decades, some of these signaling pathways and their related adaptor molecules and proteins have been introduced as valuable targets for the treatment of ALL and other solid and hematopoietic cancers. In this study, we evaluated the effects of two small molecule inhibitors, venetoclax and ibrutinib, on the immune evasion mechanisms in patients with ALL. The impacts of both SMIs were investigated on the proliferation capacity, apoptosis rate and expression of the immune checkpoint ligands in primary leukemic B-cells isolated from ALL patients. Considering lowering proliferation capacity, our results demonstrated a significant superiority of venetoclax and ibrutinib over vincristine. This result, as shown before, can be because of the cell cycle arrest at the G0/G1 phase and the suppression of the cell proliferation genes cyclin D1 and E2F1 by venetoclax and ibrutinib [[21], [22], [23], [24]]. We also found an increasing trend in cellular apoptosis rate, which was more remarkable for venetoclax compared to ibrutinib and vincristine. This finding can be explained by the differences in the cell death mechanisms applied by these two SMIs. Venetoclax directly induces cell death by inhibiting Bcl-2, which is an anti-apoptotic protein, and ibrutinib promotes apoptosis through triggering endoplasmic reticulum (ER) stress-induced cell death, another programmed cell death mechanism. There is another possible reason for low apoptosis rate induced by BTK inhibitors. The ER operates best under a specific balance of redox homeostasis and with a high concentration of calcium ions (Ca2+) within its lumen. However, when Ca2+ is delivered at inappropriate times or locations, it can trigger cell death through a process induced by ER stress, caspase activation, and finally apoptosis [[25], [26], [27], [28]]. Store-operated Ca2+ entry (SOCE) is a major mode of Ca2+ uptake in cells which can contribute to bringing Ca2+ from behind the phospholipid membrane to the intracellular space. Therefore, initiation of apoptosis can most probably be blocked by hindering SOCE, followed by restricting calcium entry into the ER [27,29]. Also, according to the previous studies, Bcl-2 plays a role in modulating SOCE, potentially preventing apoptosis triggered by ER stress [27,30]. So, high expression of Bcl-2 in leukemic cells can impede the ER stress-induced apoptosis which can be a possible reason for the non-significant apoptosis rate induced by BTK inhibitor alone. Taking these considerations into accounts, it can be postulated that combination therapies using ibrutinib with Bcl-2 inhibitor can increase its therapeutic efficacy.

Expression of immune checkpoint ligands and anti-inflammatory cytokines are considered as some of the most important immune evasion mechanisms in tumor cells. Naturally, immune checkpoint molecules are found on the surface of healthy adoptive immune cells. Interactions between these molecules and their specific ligands expressed on the surface of leukemic cells, can inhibit the anti-tumor activities of immune system, but the exact intracellular signaling pathways and molecular mechanisms are not clear yet. According to the previous studies, ALL blasts can elevate the secretion of TGF-β and expression levels of PD-L1, Gal-9, CD47, CD155, and CD200 [[15], [16], [17], [18], [19]]. Therefore, we evaluated the expression levels of these molecules after treatment with Bcl-2 and BTK inhibitors. Despite non-significant ligand expression alterations, we could identify subtle changes. All treated groups showed an increasing trend in CD47, which was more notable for venetoclax and vincristine. This result was somewhat expected. Although venetoclax is shown to be effective in inducing apoptosis and preventing tumor proliferation, it can induce the CD47 expression on cancer cells. CD47 is a ligand that can be expressed on ALL blast cells to prevent phagocytic activity, through a “Don't Eat Me” signal [[31], [32], [33]]. According to the studies by Claire Pluchart et al. [34] and Miaomiao Li et al. [35], vincristine and venetoclax can trigger the phosphatidylserine exposure to the outer layer of the cell membrane, which transmits an “Eat Me” signal. So in order to counter with this action, tumor cells start to express more CD47 lignads on their surface [[34], [35], [36]]. These explanations may suggest adding a CD47 inhibitor to the combination treatment protocols with the mentioned drugs. Morever, Shimada et al. suggested in their study that TGF-β level can be increased with CD47 silencing which shows TGF-β signaling is regulated by CD47 [37]. Thus, no significant difference for TGF-β in our experiments can be explained by the increasing in the CD47 level. Also, no significant change was observed in PD-L1 mRNA expression after treatment of leukemic cells with SMIs. According to the Gaber et al.'s study on ALL patients, PD-L1 expression has a varied range on ALL leukemic blasts [38]. The reason for the lower expression of PD-L1 in ALL leukemic cells may be because of a transcription factor necessary for the establishment of germinal centers, BCL6. It has been demonstrated that B-ALL is significantly influenced by BCL6, specially those with the t(1; 19) translocation, and elevated BCL6 mRNA levels was observed in B-ALL patients [39,40]. It has been shown in germinal center B cells that BCL6 is a crucial negative regulator of PD-L1 and PD-L2 which PD-L1 is upregulated when BCL6 is deleted in germinal center B cells [41]. Consequently, the lack of an alteration in PD-L1 expression following treatments can be explained by the high expression of BCL6 in ALL leukemic blasts. Regarding CD200, ibrutinib demonstrated an elevation and venetoclax showed a decreasing in its mRNA expression; however, the outcomes did not reach statistical significance. It should be noted that inconsistencies have been reported about the expression levels of CD200, CD155, and Gal-9 across different studies. The findings of a clinical trial conducted on chronic lymphocyte leukemia patients revealed that the expression level of CD200 was diminished after receiving of ibrutinib [42]. Our results revealed in the presence of ibrutinib, there was a modest upregulation in the level of Gal-9 mRNA and a slight downregulation in CD155 expression. Taghiloo et al. showed in a murine model of acute myeloid leukemia (AML) that treatment with a dual PI3K/mTOR inhibitor and TLR-7/8 agonist can reduce the expression of PD-L1, Gal-9, and CD155 [43]. Soltanshahi et al. conducted a study to examine the impact of ibrutinib on the levels of CD155 and Gal-9 in MCF-7 breast cancer cells. Their findings revealed a notable reduction in CD155 expression, while no alterations were detected in the expression of Gal-9 [44]. In another report by Taghiloo et al. on HL-60 AML cell line, the expression of PD-L1, Gal-9, and CD155 was more effectively suppressed when the PI3K, Akt, and mTOR inhibitors were combined [8]. The immunological relevance of SMIs interaction with immune checkpoints received increasing attention during the last years, and ongoing studies increasingly direct their interest in the therapeutic appeal of combining immune checkpoint blockers and small molecule inhibitors to achieve synergy in antitumor activity for hematological tumors. Also, in syngeneic mice tumor models, Kohlhepp et al. have shown that venetoclax can improve the anticancer efficacy of immune checkpoint inhibitors [45]. Similarly, in a mouse model of CLL, Hanna et al. demonstrated that ibrutinib plus antibodies that disrupt the PD-1/PD-L1 axis enhanced CD8^+^ T cell effector activity [46]. The variations in the tumor microenvironment as well as differences in the methodological approaches, may be as important causes of the expression differences observed between these in vitro, in vivo, and clinical trial studies. While there was no tumor microenvironment in our investigation and isolated ALL leukemic cells were directly exposed to the drugs, it should be considered that in in vivo and clinical trial studies, the tumor microenvironment comprising various cells which can influence the immune evasion mechanisms and the expression of immune checkpoint molecules. Genetic heterogeneity and possibly limited number of patient samples may be other explanations for these differences.

## 5 Conclusion

Briefly, the in vitro treatment of isolated ALL leukemic cells with ibrutinib, venetoclax, and vincristine resulted in a decreasing in cell proliferation and an increasing in apoptosis rate, which was more remarkable for venetoclax. Moreover, alterations in the expression of genes related to the immune checkpoint inhibitory ligands and anti-inflammatory cytokine were observed. Consequently, it can be inferred that small molecule inhibitors not only hinder proliferation and enhance apoptosis, but also affect the expression of inhibitory immune checkpoint ligands. By elucidating the precise underlying mechanisms, these drugs could emerge as promising novel therapeutic options, particularly in the context of combination therapy for ALL and other tumors. Nevertheless, further investigations are warranted to validate and elucidate the intricate mechanisms.

## Authors’ Contribution

Armin Dozandeh-Jouybari performed the main experiments and wrote the initial draft of the manuscript. Fatemeh Mousavi-Mirkalaei consulted on the optimizatopn of the experiments. Saeid Taghiloo participated in the primer desgining and final data analysis. Hossein Karami, Mohammad Naderisorki, Ehsan Zaboli and Mohammad Eslami-Jouybari participated as clinicians in the sample collection and ALL diagnosis. Tohid Kazemi consulted on the optimizatopn of the experiments. Hossein Asgarian-Omran conceived the original idea, designed the experiments, edited and approved the final manuscript.

## Data reproducibility

The dataset presented in the study is available upon request from the corresponding author during submission or after its publication.

## Funding/support

This study was financially supported by 10.13039/501100004160Mazandaran University of Medical Sciences (grant no: 9549) and Tabriz University of Medical Sciences (grant no: 71033).

## Declaration of competing interest

The authors declare that they have no known competing financial interests or personal relationships that could have appeared to influence the work reported in this paper.

## Data Availability

Data will be made available on request.
